# Thin Film Electrodes for Anodic Stripping Voltammetry: A Mini-Review

**DOI:** 10.3389/fchem.2021.809535

**Published:** 2022-02-02

**Authors:** Bryan R. Wygant, Timothy N. Lambert

**Affiliations:** Sandia National Laboratories, Albuquerque, NM, United States

**Keywords:** anodic stripping voltammetry (ASV), metal ion analysis, batteries and energy applications, medical applications, environmental applications, metal thin film electrodes

## Abstract

Anodic stripping voltammetry (ASV) is a powerful electrochemical analytical technique that allows for the detection and quantification of a variety of metal ion species at very low concentrations in aqueous media. While early, traditional ASV measurements relied on macroscopic electrodes like Hg drop electrodes to provide surfaces suitable for plating/stripping, more recent work on the technique has replaced these electrodes with thin film metal electrodes generated *in situ*. Such electrodes are plated alongside the analyte species onto the surface of a primary electrode, producing a composite metal electrode from which the analyte(s) can then be stripped, identified, and quantified. In this minireview, we will explore the development and use of these unique electrodes in a variety of different applications. A number of metals (e.g., Hg, Bi, Sn, etc.) have shown promise as thin film ASV electrodes in both acidic and alkaline media, and frequently multiple metals in addition to the analyte of interest are deposited together to optimize the plating/stripping behavior, improving sensitivity. Due to the relatively simple nature of the measurement and its suitability for a wide range of pH, it has been used broadly: To measure toxic metals in the environment, characterize battery materials, and enable biological assays, among other applications. We will discuss these applications in greater detail, as well as provide perspective on future development and uses of these thin film electrodes in ASV measurements.

## Introduction

**SCHEME 1:**
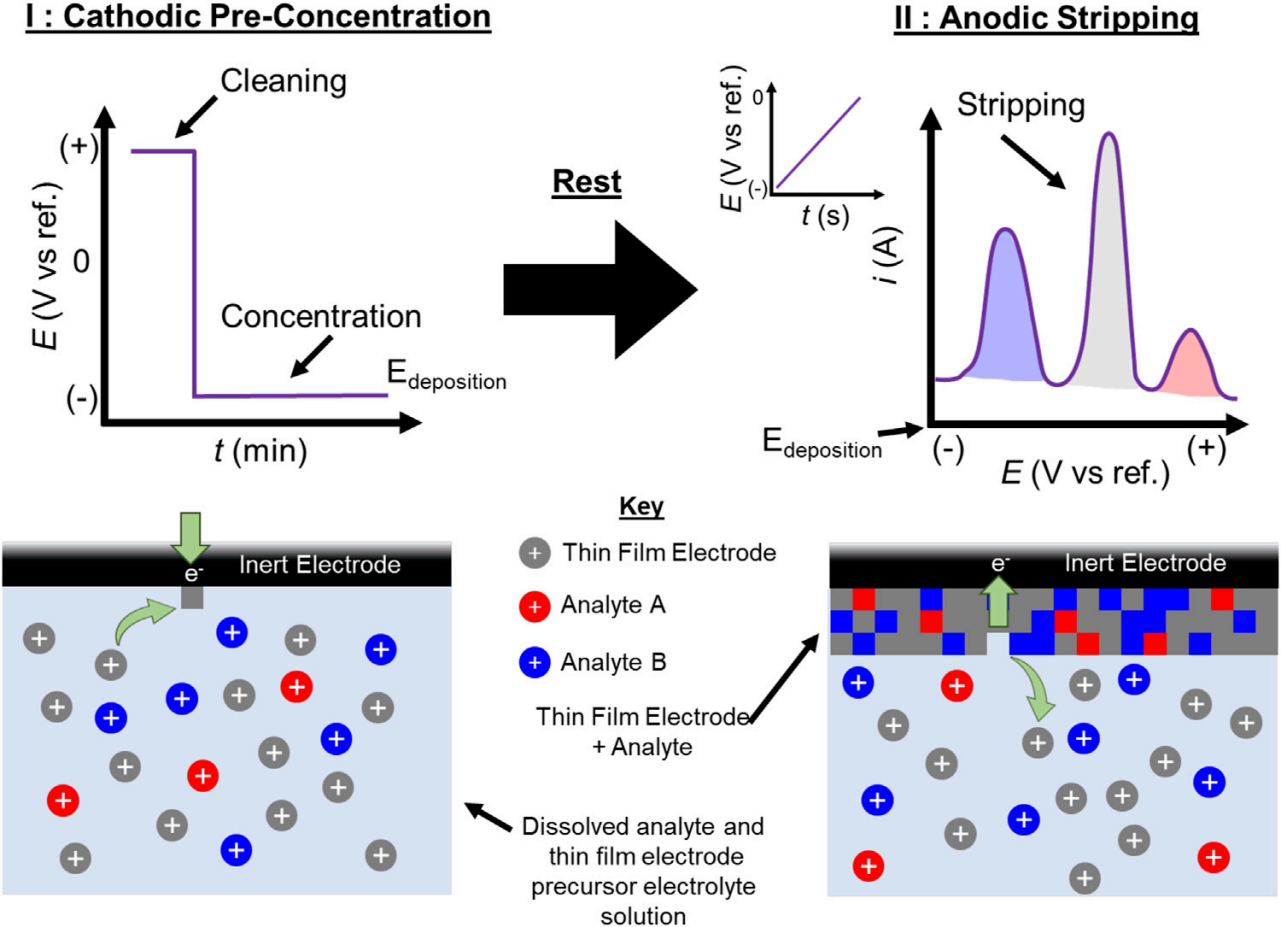
Graphical depiction of a typical ASV experiment using an *in situ* electrode. During pre-concentration (I) an oxidizing current is first applied to clean the electrode surface, before a reducing potential sufficient to electroplate the soluble ionic metal electrode and the analyte species (E_deposition_) is applied. After a brief rest, the potential is swept positive from E_deposition_, anodically stripping (II) the electrode and analyte species from the inert electrode surface. Peak potentials from the stripping can be used to identify the species, and the integrated charge under each peak can be related to the concentration of each analyte in solution.

Anodic stripping voltammetry (ASV) is an important electrochemical technique with a history stretching back to the earliest days of electrochemistry(Zbinden, 1931). A simple technique, it allows for both the identification and quantification of electroactive species in solution at nanomolar (nM) concentrations (10s of ppb) and lower and has sensitivities that are competitive with techniques like inductively coupled plasma mass spectrometry (ICP-MS)(Bard and Faulkner, 2001; Duay et al., 2017a). A detailed description of the process is available elsewhere (Ellis, 1973; Bard and Faulkner, 2001; Borrill et al., 2019), but a brief overview of the technique is provided in Scheme 1. ASV consists of two primary steps: 1) A cathodic electrodeposition to concentrate dissolved analytes at an electrode and 2) anodic stripping of the electrode to re-dissolve the species into solution. Each electrodeposited species should result in a stripping peak, where the voltage of each peak can be used to identify the species being oxidized and the peak current/integrated charge is related to the concentration of the species in solution.

ASV is traditionally performed using metallic Hg electrodes, generally in the form of hanging mercury drop electrodes (HMDE) or mercury film electrodes (MFE)(Ellis, 1973; Bard and Faulkner, 2001). Both electrodes benefit from the high conductivity of Hg, the ability of Hg to form easily-stripped amalgams with many metals, and the poor hydrogen evolution reaction (HER) kinetics on Hg that allow for more reductive (positive) potentials to be applied during electrodeposition. MFEs are typically preferred over HMDEs due to their lower electrode volume and thickness, both of which increase the sensitivity of the measurement(Bard and Faulkner, 2001). To achieve low electrode thickness, MFEs are typically co-deposited onto an inert electrode, typically glassy carbon, from the analyte solution after adding small quantities of Hg^2+^(Bard and Faulkner, 2001; Borrill et al., 2019). Co-deposition also simplifies sample preparation and reduces the quantity of Hg required. Although these properties make MFEs attractive for use in ASV analyses and reduce the quantity of Hg needed, the inherent toxicity and significant health risks posed by Hg has led researchers to search for more benign alternatives.

As a result, recent work in the field has focused on replacing Hg with metals such as Bi (Wang et al., 2000; Wang et al., 2001; Li et al., 2010; Duay et al., 2017a), Sn (Czop et al., 2011; Li et al., 2012), Au (Bu et al., 2015; Kogan et al., 2018), and others(Tyszczuk et al., 2007; Tesarova et al., 2009; Duay et al., 2017b). Although these metals do not form the same amalgams that Hg does, their low toxicity and compatibility with non-acidic electrolytes has made them an exciting addition to the field. Additionally, most can be co-deposited like Hg to form metal thin film electrodes (MTFEs) that are very similar to more traditional MFEs. In this mini review, we will provide a brief overview of the development and application of these MTFEs in ASV analyses and provide a perspective on future directions for such electrodes moving forward.

## Electrode Types

### Mercury Film Electrodes

ASV using MFEs came into common use during the 1950s and 1960s (Ellis, 1973) as an accurate electrochemical technique for measuring metal concentration in aqueous samples(Batley and Florence, 1974). While MFEs allow for high sensitivity ASV measurements, the high concentration of analyte metal in the electrode can also result in interference from intermetallic phases. To improve the reproducibility of the measurements, MFEs are typically deposited onto glass carbon (GC) electrodes. GC electrodes are highly conductive and can be polished to a very smooth surface that is impermeable to and chemically unreactive with Hg, allowing for the creation of a similar electrode surface over multiple experiments. Although they can be deposited from a separate Hg^2+^ solution and then used for ASV analysis (*ex situ* electrode), MFEs are frequently deposited *in situ* with the analyte species during the pre-concentration step. The result is a heterogeneous film tens of microns thick composed of the MFE and the analyte(s) of interest that can be readily stripped and has a high quantity of analyte to improve detection(Bard and Faulkner, 2001).

Although frequently used for testing metal concentration in aqueous samples, MFEs can also be used for ASV analysis of nonaqueous samples. For example, Marques *et al.* used an *ex situ* MFE electrode to determine the concentration of Pb and Cu in biodiesel(Martiniano et al., 2013). The researchers used 1-propanol to create a microemulsion of biodiesel in nitric acid electrolyte, allowing Pb and Cu contaminants in the organic phase to be plated at the MFE. They were able to demonstrate low levels of detection (LoD) for both Pb and Cu (2.91 and 4.69 nM, respectively) and showed linear concentration ranges of 20–100 nM for both species. Similar results were obtained by de Oliveira *et al.* who used a similar MFE to measure the Zn, Cu, Pb, and Cd content of fuel ethanol generated from biological sources(De Oliveira et al., 2004). MFEs can even be used for ASV measurements of non-metallic analytes, as demonstrated by Huan and coworkers when quantifying S^2−^ in synthetic wastewater(Huang et al., 2010). Cd^2+^ and S^2−^ react spontaneously in solution to form CdS, and so by measuring the concentration of soluble Cd^2+^ using an *ex situ* MFE before and after the addition of a sample containing S^2−^, the quantity of Cd^2+^ consumed (and thus the quantity of S^2−^ added) could be calculated. The measurement proved to be more sensitive than competing techniques (LoD = 10.3 nM S^2−^) and had a linear range of 20–220 nM. Taken together, these examples showcase the utility of MFEs for ASV analysis of nontraditional analytes and from nontraditional sources.

### Non-Hg MTFEs

Although MFEs are nearly ideal electrodes for ASV analysis, concerns over Hg toxicity and a desire to continue to improve the technique has driven the development of a thriving field of MTFEs composed of other metals. First reported by Wang *et al.* (Wang et al., 2000), Bi is one of the most popular non-Hg MTFEs and can be used in a manner similar to that of both *in* and *ex situ* Hg analogs. Wang found that the addition of Bi^3+^ to the deposition solution was essential to the plating of Zn, Cd, and Pb, and that it could be used in concert with an MFE to improve sensitivity to Tl and Cd(Wang et al., 2000). Later reports from Wang *et al.* also showed that, like MFEs, Bi MTFEs could be plated *ex situ* and used for ASV determination of Pb concentration(Wang et al., 2001). The electrode allowed for a Pb^2+^ LoD of 1.4 nM and provided a linear range of 0–483 nM, opening the door to easily disposable Bi MTFEs for incorporation into low-cost electrodes for ASV. These early results matched or exceed similar results from MFE analyses (Wang et al., 2000), and demonstrated the utility of non-Hg MTFEs for use in ASV analysis.

Further research showed that Bi electrodes could also be used to perform ASV analysis in highly alkaline solutions(Li et al., 2010). Typical ASV analysis takes place in acidic media where the various metal ions exist in a solvated *M* ^*+*^ state, and should follow well-established mechanisms for the electrodeposition of metal in acidic media(Borrill et al., 2019). In solutions where pH > 11, however, Bi^3+^ can complex with the large concentration of OH^−^ in solution to form soluble, easily stripped/plated Bi(OH)^2+^ species(Li et al., 2010). This hydroxide complexation property is shared by other metals like Pb and Zn (Duay et al., 2017a; Duay et al., 2017b), while Hg forms insoluble hydrated mercury oxides that prevents the use of MFEs(Li et al., 2010). Using a Bi MTFE in 0.1 M NaOH, prior work has shown it is possible to achieve an LoD of 1.93 nM for Pb with a linear range of 9.6–290 nM (Li et al., 2010), matching the level of sensitivity for measurements in an acidic electrolyte and demonstrating the benefits of Bi MTFEs over MFEs for ASV analysis in alkaline conditions.

Building on this work, other metals have likewise been used as MTFEs for ASV analysis, among them Cu (Jovanovski et al., 2015; Jovanovski and Hrastnik, 2019; Hrastnik et al., 2020), Ga (Copeland et al., 1974; Tyszczuk et al., 2007; Mc Eleney et al., 2020), Pb (Duay et al., 2017b; Arnot and Lambert, 2020), Sb, (Hocevar et al., 2007; Tesarova et al., 2009; Slavec et al., 2010; Ruengpirasiri et al., 2017), Sn (Zhu et al., 2007; Czop et al., 2011; Li et al., 2012; Kokkinos et al., 2018), and precious metals like Au and Ag(Ochab et al., 2017; Stojanović et al., 2018; Pollok et al., 2020). A brief overview of the various MTFE metals, analytes, and sensitivities is included in Table 1. While used less frequently than Hg or Bi, they are generally employed when one of these more common metals is unsuitable for the assay. For example, Pb allows for measurements of Cu and Bi in highly alkaline solutions (e.g., 35 wt% KOH) (Duay et al., 2017b; Arnot and Lambert, 2020), while Ga films can be used to reduce Cu interference while studying Zn (Copeland et al., 1974). The general trend in the field has been one of diversification of MTFE choice to enable higher quality ASV analyses, both by using new metals and through the addition of metal additives to the electrodes. Ga is one such additive and has been used in both MFEs and Bi MTFEs to improve their Zn sensitivity when Cu also present (Tyszczuk et al., 2007). Ga reacts readily and selectively with Zn during pre-concentration to form a single Ga-Zn species, preventing the formation of multiple Cu-Zn intermetallic species and simplifying the ASV assay for Zn (Copeland et al., 1974).

**TABLE 1:** Overview of MTFEs metals, analytes, and sensitivities.

Electrode metal	pH range	Analytes	LoD	Linear range
Hg	3–9	Pb^1^	2.91 nM	20–100 nM
		Cu^1^	4.69 nM	20–100 nM
		Cd^2^	9.70 nM	10–100 nM
		Zn^2^	8.06 nM	10–100 nM
		S^3^	10.3 nM	20–220 nM
Bi	3–5, >11	Pb^4^ (acidic)	1.40 nM	0.480 nM
		Pb^5^ (basic)	1.93 nM	10–290 nM
		Cd^6^	2.72 nM	9–1,335 nM
		TI^7^	0.021 nM	0.05–5 nM
		Sn^8^	16 nM	210–2,100 nM
		Pd^9^	103 μM	188–940 μM
		Zn^10^	24.5 μM	46–306 μM
Cu	3–9	Hg^11^	0.50 μM	50–500 μM
		Pb^11^	0.29 μM	24–338 μM
		Ni^12^	3.15 μM	17–170 μM
Ga	3–5	Zn^13^	12 nM	50–2000 nM
Pb	>11	Cu^14^	10.5 μM	16–126 μM
		Bi^15^	40.7 nM	48–480 nM
Sb	2–5	Cd^16^	3.23 μM	118–1,246 μM
		Pb^16^	4.34 μM	97–676 μM
		Cu^17^	15.7 μM	79–2,360 μM
Sn	3–5	Cd^18^	9.79 μM	890–890 μM
		Cr^18^	38.46 μM	192–1920 μM
		Zn^19^	13.8 μM	0–3,059 μM
Au	2–7.5	Se^20^	0.85 nM	5–10 nM
		Ag^21^	2.6 fM	3–337 fM
Ag	3–7	Cr^22^	0.1 μM	0.35–40 μM

1 (Martiniano et al., 2013).

2 (De Oliveira et al., 2004).

3 (Huang et al., 2010).

4 (Wang et al., 2001).

5 (Li et al., 2010).

6 (Jiang et al., 2018).

7 (Rutyna and Korolczuk, 2014).

8 (Prior, 2010).

9 (Rosolina et al., 2016).

10 (Duay et al., 2017a).

11 (Jovanovski et al., 2015).

12 (Hrastnik et al., 2020).

13 (Tyszczuk et al., 2007).

14 (Duay et al., 2017b).

15 (Arnot et al., 2021).

16 (Hocevar et al., 2007).

17 (Slavec et al., 2010).

18 (Zhu et al., 2007).

19 (Czop et al., 2011).

20 (Ochab et al., 2017).

21 (Kogan et al., 2018).

22 (Stojanovic et al., 2018).

### Experimental Considerations for ASV

As there are many metals suitable for use as ASV MTFEs, care must be taken when choosing an electrode material. First and foremost, regardless of the metal used to create the MTFE it is important to consider complications inherent to the use of thin film electrodes. These include, but are not limited to, the cleaning of the substrate/electrode surface (before electrode deposition and/or each experiment), unavoidable fouling of electrode from contaminant species in solution, the solubility of metallic species in solution, film-to-film variations of *in situ* MTFEs, and proper calibration of the assay prior to measurements. The last two points are of particular importance, as the low quantities of metal used to create the MTFEs could easily result in uneven films, and therefore electrochemical responses, that must be taken into account. For a more detailed discussion of these topics, we recommend the tutorial review written by Borrill et al. (Borrill et al., 2019) After these general points, the next consideration is the pH of the solution the assay will be performed in. If there is sufficient electrolyte concentration, regardless of pH, ASV analysis can be performed in the native sample solution with minimal preparation, unlike inductively coupled plasma mass spectrometry (ICP-MS) (Duay et al., 2017a). Sb MTFEs have been shown to be effective Hg replacements in highly acidic media (pH < 2) (Tesarova et al., 2009), but ASV is frequently performed in moderately acidic solutions (pH 3–5) to prevent hydrolysis by the metal ions in solution. Such conditions are suitable for traditional MFEs, as well as Bi, Cu, Ga, Sb, and Sn MTFEs (Wang et al., 2000; Tyszczuk et al., 2007; Tesarova et al., 2009; Li et al., 2012; Jovanovski et al., 2015). For solutions of pH 7-9, Hg and Cu are commonly employed (Guell et al., 2008; Hrastnik et al., 2020). For highly alkaline solutions (pH > 11), both Bi and Pb form soluble hydroxide complexes and have wide electrochemical windows that make them ideal MTFEs (Li et al., 2010; Arnot and Lambert, 2020).

After pH, one should also consider the chemical interactions of the species of interest with the electrode and other species in solution. Bi and Hg have wide electrochemical windows and good plating behavior that make them ideal electrodes for many other metals (Wang et al., 2000). Even for Bi and Hg MTFEs, some analyte species may deposit multiple intermetallic phases when plating or suffer from poor plating efficiency, complicating the assay and requiring additive species to reduce the impact. As mentioned before, Ga prevents the formation of undesired intermetallic phases in the presence of Cu (Copeland et al., 1974), while cetyltrimethylammonium bromide (CTAB) has been found to reduce the interference of Pb on Sn assays (Prior, 2010). With regard to plating efficiency, a combination of Pb and Cd additives has been shown to improve the plating of both Zn and Pd using Bi MTFEs (Rosolina et al., 2016; Duay et al., 2017a). Based on these results, we speculate that there may be a wide range of metals that are suitable for use as additives to ASV MTFEs.

## Review of Applications

**FIGURE 1:**
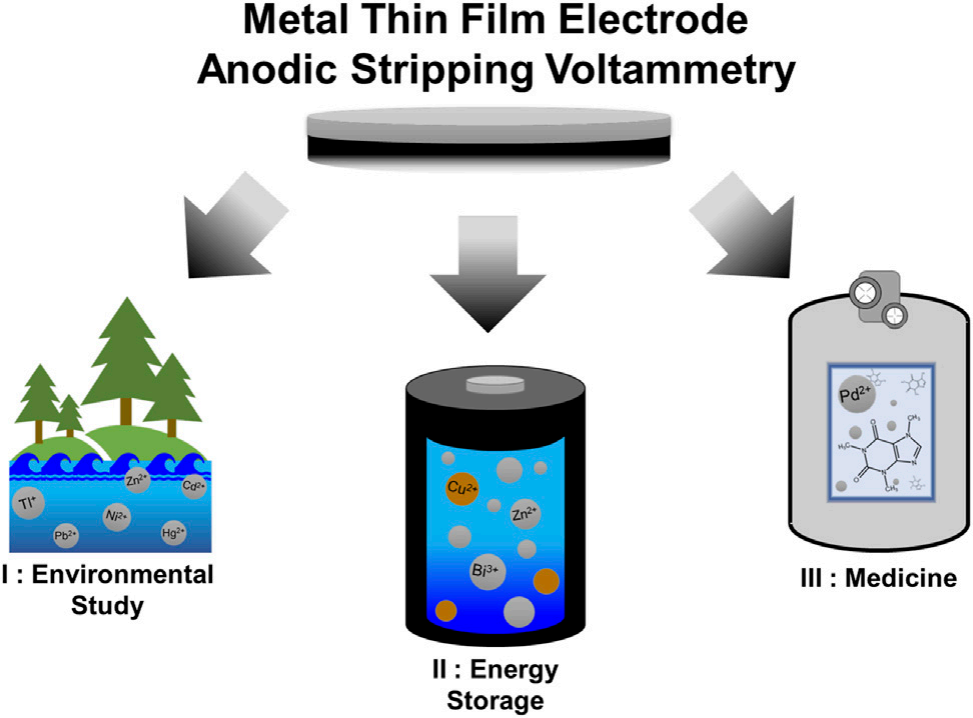
Overview of the applications of MTFE-based ASV analysis, including I) identification and quantification of toxic metals in the environment, II) quantification of metal species in alkaline battery systems, and III) detection of catalysts and other metals in medically important systems.

The relative simplicity and broad applicability of MTFEs and ASV assays has led to their use in a wide range of fields, including the monitoring of various metals in the environment (Kefala, 2003; Lee et al., 2017; Lu et al., 2018), the quantification of soluble metal species in battery electrolytes (Duay et al., 2017a; Duay et al., 2017b; Arnot and Lambert, 2020), and even measuring the quantity of biomolecules in blood serum (Pollok et al., 2020). Below, we have provided an overview of some recent applications of MTFEs and ASV in these fields and others (Figure 1).

### Environmental Studies

Because ASV requires significantly less sample preparation and equipment to accurately quantify metals in a wide range of sample media, it is frequently used to detect and measure the concentration of various heavy metals (Pb, Cd, Zn, Tl, Ni, etc.) in the environment (Lu et al., 2018). As an example, Rutyna *et al.* used an *in situ* Bi MTFE to measure the concentration of Tl in samples of lake water, achieving an LoD of 0.021 nM for Tl with a linear range of 0.05–5 nM (Rutyna and Korolczuk, 2014). This level of accuracy required a second preconcentration step be added to the procedure, significantly concentrating the Tl present in the solution and allowing for detection at ultratrace concentrations, even in complex media.

Similarly, Jian *et al.* showed that a Bi MTFE deposited onto GaN was able to achieve a 2.72 nM LoD for Cd with a linear range of 8.9–1,334.5 nM (Jiang et al., 2018). Here, GaN provided an inert, highly conductive substrate upon which the Bi MTFE could be deposited during the ASV assay, and the composite electrode showed high Cd recovery efficiency from several complex matrixes including natural waters and others. As a final example, Lee *et al.* used a 3D printed stainless steel electrode to create an *ex situ* Bi MTFE for ASV measurements of Cd and Pb (Lee et al., 2017). The researchers demonstrated that custom electrodes could be created and successfully applied to electrochemical measurements, achieving LoDs of 17 and 83 nM for Pb and Cd, respectively, and linear ranges between 200 and 3,000 nM. While just a few examples, they demonstrate the use of MTFEs and ASV in detecting a range of toxic metals in environmentally relevant sample matrixes.

### Batteries and Energy Applications

ASV has also been useful in the study and characterization of alkaline battery systems, recently in the analysis of metal ion diffusion through polymer separators of use in Zn/MnO_2_ batteries (Duay et al., 2017a; Duay et al., 2018; Kolesnichenko et al., 2020; Arnot et al., 2021). During electrochemical cycling, soluble Zn species generated at the Zn anode can react with the MnO_2_ cathode, irreversibly forming ZnMn_2_O_4_ and decreasing the capacity of the cell (Duay et al., 2017a). In addition to preventing electrical short circuits, Zn-impermeable separators can prevent this reaction, but identifying promising polymers through traditional ICP-MS crossover experiments is time and processing intensive. Recently, our group has shown that ASV using MTFEs can replace ICP-MS and significantly simplify measurements of Zn permeability, and have used the assay to characterize several commercial and custom battery separators (Duay et al., 2017a; Kolesnichenko et al., 2020). Using *in situ* Bi MTFEs with Pb and Cd additives, Duay et al*.* achieved an LoD of 24.5 μM for Zn in a 35 wt% solution of KOH, with a linear response range from 45.8–305.9 μM (Duay et al., 2017a). This result was similar to the LoD of ICP-MS (115 μM) in the same KOH solution, but could be performed in the concentrated alkaline electrolyte, whereas the ICP-MS required significant dilution and neutralization.

In subsequent studies, our group also demonstrated that *in situ* Pb MTFEs could be used to measure diffusion of Cu and Bi through separators; both are promising additives for Zn alkaline batteries. Again working directly in the 35 wt% KOH solution used in alkaline batteries, a Pb MTFE achieved LoDs of 10.5 μM and 40.7 nM for Cu and Bi, respectively, with linear responses between 15.7–125.9 μM for Cu and 47.8–478.5 nM for Bi (Duay et al., 2017b; Arnot and Lambert, 2020). Although the use of MTFE-based ASV analysis in battery research is in its infancy, we believe these results indicate it has great promise and will discuss them at more length in the Future Outlooks.

### Medical Applications

The final application of MTFEs for ASV pertains to medical assays, where the sensitivity and tolerance of these electrodes for complex matrices makes them ideal for detecting medically relevant species. As an example, Rosolina *et al.* used an *in situ* Bi MTFE to quantify Pd levels in simulated pharmaceutical matrixes(Rosolina et al., 2016). Possessing some toxicity, Pd is used to catalyze many organic reactions during drug synthesis and trace quantities can be left in the final consumer product. Without requiring time-consuming digestion steps, the authors demonstrated an LoD of 103 μM in an aqueous solution containing caffeine, with a linear range of 188–939.4 μM. Interestingly, the authors noted that Cd and Pb improved the sensitivity of the measurement for Pd in a manner analogous to that reported by Duay *et al.*(Duay et al., 2017a) This result suggests that these species may be important in achieving low LoDs with Bi MTFEs.

MTFEs have also been used to quantify organic species like quinolones and peptides using ASV (Kergaravat et al., 2018; Pollok et al., 2020). Kergaravat *et al.* used an *ex situ* Bi MTFE to quantify two separate quinolones in both buffer solutions and cladoceran crustaceans culture media; they reported an LoD of 1 nM for moxifloxacin and other quinolones with a linear range of 2.99–87.2 nM (Kergaravat et al., 2018). This allowed the researchers to use ASV to show a correlation between degradation of the molecule and the death of the crustaceans, indicating that similar processes may occur in nature. To measure complex heart-failure related peptides, the Crooks group has coupled Ag MTFEs with peptide-specific antibodies for N-terminal prohormone brain natriuretic peptide (NT-proBNP) (Kogan et al., 2018; Pollok et al., 2020). During the assay, sequential stripping of both an *ex situ* Au MTFE and galvanic exchange to produce an *in situ* Ag MTFE that can be used to determine the quantity of the peptide in solution. The Ag nanoparticles are bound to peptide-specific antibodies, creating the correlation between the quantity of Ag measured during ASV and the quantity of peptide present in the sample. Using this assay, Pollok *et al.* achieved an LoD near 0.58 nM and a linear range of 0.58–2.33 nM (Pollok et al., 2020). Although this sensitivity is currently insufficient for clinical application, the whole assay can be performed on a paper-based device that promises to reduce the costs associated and complex equipment typically associated with these types of assays.

The above examples, while only a small selection of the many applied MTFEs in literature, represent a variety of applications where MTFE-based ASV is found. They showcase the sensitive measurements and chemical selectivity that MTFEs bring to electrochemical analysis and the diverse applications that such relatively simple systems can be used in, making a compelling case for the inclusion of MTFEs and ASV in the standard analytical toolbox.

## Future Outlook

Although the previous examples show how mature MTFEs and ASV have become, better characterization of the MTFEs used to perform ASV analysis is still needed. MTFEs are frequently electrodeposited and stripped without ever being removed from solution, precluding any physical or chemical characterization with techniques like scanning electron microscopy (SEM) or X-ray photoelectron spectroscopy (XPS). Doing so means that while great care is frequently taken to optimize the deposition parameters of an assay (MTFE precursor concentration, analyte concentration, deposition potential, etc.), we do not know how these optimizations relate to the morphology or chemistry of the resulting electrode surface.

Demonstrating the importance of these properties, Rajamani et al. performed a systematic study of Bi electrodeposition using different deposition conditions and solutions additives to determine how each impacted the final morphology, chemistry, and physical properties of the MTFE (Rajamani et al., 2016). They found that both additives and deposition conditions can impact the properties of the deposited MTFE, and that small, smooth Bi crystallites show better Pb sensitivity than higher surface area crystallites. Studies like this illustrate that a holistic understanding of MTFEs can produce better electrodes for ASV analysis and reinforce the need for more such research.

Beyond electrode characterization, additional work developing non-toxic MTFEs would also be of benefit. While Bi is now widely recognized as a suitable replacement for Hg, recent work has shown that the addition of toxic metals like Pb and Cd can improve the sensitivity of these otherwise non-toxic MTFEs (Rosolina et al., 2016; Duay et al., 2017a). With work, less-toxic alternatives like Ga or Sn may be able to replace these important additives. Such work may also lead to MTFEs with improved ASV performance in neutral and alkaline electrolytes, opening doors to new applications for the technique. New fields of study could include electrochemical fuel cells, where electrocatalytically active metals like Pt are known to dissolve and redeposit in the alkaline electrolytes (Deng et al., 2019). As in the Zn assays performed by our group to evaluate alkaline battery separators (Duay et al., 2017a; Duay et al., 2018; Kolesnichenko et al., 2020; Arnot et al., 2021), Sn or Bi MTFEs may be useful in quickly and simply quantifying this dissolution. Alternatively, MTFE-based ASV analysis like that demonstrated by Crooks and coworkers could find expanded use in electrochemical assays of other biomolecules. ASV analysis is highly sensitive and relatively simple to perform, making it an exciting option for inexpensive, portable assays of many biologically important species. Finally, recent work has demonstrated that multi-metal or alloy MTFE electrodes show improved ASV performance when compared to single metal electrodes (Rosolina et al., 2016; Duay et al., 2017a; Valera et al., 2018). These studies and others demonstrate that metal alloys can exhibit increased HER overpotentials and improved deposition/stripping behavior when compared to electrodes composed of a single metal, but additional work is needed to optimize and characterize these electrodes. Determining how the addition of a specific metal impacts electrode performance will be important, as well as whether the resulting MTFE is better classified as a bimetallic electrode or an alloy, will allow for more directed development of these MTFEs in the future.

## Conclusion

MTFEs offer many benefits for ASV analysis over more traditional HMDE and bulk electrodes. MTFEs can be deposited *in situ* along with the analyte species, producing highly concentrated electrode films with excellent sensitivity over broad concentration ranges. Many metals can be used to create MTFEs, including both the traditional (but toxic) Hg and less toxic alternatives like Bi or Sn. This breadth allows for the tailoring of the MTFE for a particular assay, producing electrochemically distinct bimetallic alloys with the species of interest, even in the presence of other species of interest. Coupled with the relative ease of ASV sample preparation, these electrodes allow for higher throughput analysis of samples ranging from natural water to blood serum. MTFE-based ASV assays have been applied to many different systems in the recent past, and should remain a sensitive and useful tool for chemical analysis in these, and other, applications moving forward.
